# Physical exercise and cardiovascular response: design and implementation of a pediatric CMR cohort study

**DOI:** 10.1007/s10554-023-02950-7

**Published:** 2023-10-06

**Authors:** Meddy N. Bongers-Karmaoui, Alexander Hirsch, Ricardo P. J. Budde, Arno A. W. Roest, Vincent W. V. Jaddoe, Romy Gaillard

**Affiliations:** 1 The Generation R Study Group, Erasmus University Medical Center, PO Box 2040, 3000 CA Rotterdam, The Netherlands; 2 Department of Pediatrics, Sophia Children’s Hospital, Erasmus University Medical Center, Rotterdam, The Netherlands; 3 Department of Cardiology, Erasmus University Medical Center, Rotterdam, The Netherlands; 4 Department of Radiology and Nuclear Medicine, Erasmus University Medical Center, Rotterdam, The Netherlands; 5 Department of Pediatrics, Leiden University Medical Center, Leiden, The Netherlands

**Keywords:** Cardiovascular magnetic resonance imaging, Exercise testing, Pediatric cardiology, Population study

## Abstract

To examine feasibility and reproducibility and to evaluate the cardiovascular response to an isometric handgrip exercise in low-risk pediatric population using Cardiovascular Magnetic Resonance measurements. In a subgroup of 207 children with a mean age of 16 years participating in a population-based prospective cohort study, children performed an isometric handgrip exercise. During rest and exercise, continuous heart rate and blood pressure were measured. Cardiovascular magnetic resonance (CMR) measurements included left ventricular mass, aortic distensibility and pulse wave velocity at rest and left ventricular end-diastolic and end-systolic volumes, ejection fraction, stroke volume and cardiac output during rest and exercise. 207 children had successful CMR measurements in rest and 184 during exercise. We observed good reproducibility for all cardiac measurements. Heart rate increased with a mean ± standard deviation of 42.6% ± 20.0 and blood pressure with 6.4% ± 7.0, 5.4% ± 6.1 and 11.0% ± 8.3 for systolic, diastolic and mean arterial blood pressure respectively (p-values < 0.05). During exercise, left ventricular end-diastolic and end-systolic volumes and cardiac output increased, whereas left ventricular ejection fraction slightly decreased (p-values < 0.05). Stroke volume did not change significantly. A sustained handgrip exercise of 7 min at 30–40% maximal voluntary contraction is a feasible exercise-test during CMR in a healthy pediatric population, which leads to significant changes in heart rate, blood pressure and functional measurements of the left ventricle in response to exercise. This approach offers great novel opportunities to detect subtle differences in cardiovascular health.

**Supplementary Information:**

The online version contains supplementary material available at 10.1007/s10554-023-02950-7.

## Introduction

Cardiovascular diseases are a major public health problem and leading cause of death worldwide [1]. In adults, cardiovascular exercise tests are used as a screening tool to identify patients at increased risks of cardiovascular diseases, even when clinical symptoms are not yet present [2]. In pediatric clinical populations, cardiac exercise testing is mostly used in children with a known cardiac abnormality to evaluate the severity of the condition, effects of treatment and prognosis [3–5]. During exercise, the response of the circulatory system is designed to match the higher oxygen requirements in the exercising muscles by raising heart rate, heart contractility and blood pressure [6]. In both adult and pediatric populations with cardiac abnormalities and cardiovascular diseases, an abnormal response of the cardiovascular system to exercise is associated with poorer cardiovascular health outcomes and overall reduced quality of life [7–9].

Accumulating evidence suggests that cardiovascular diseases might originate from early life onwards [10]. Early life exposures during pregnancy and childhood may be associated with persistent cardiac structural and functional developmental adaptations, predisposing to increased risks of cardiovascular dysfunction and diseases in later life [11–13]. Recent evidence from small pediatric studies suggests that cardiovascular exercise testing may provide important information on cardiovascular health in non-diseased pediatric populations, which enables detection of subtle cardiovascular dysfunction not yet present in rest [14, 15]. The application of cardiovascular exercise tests in longitudinal birth cohort studies may serve as a valuable tool to reveal subtle cardiovascular developmental adaptations in response to early life exposures, and to better identify children who are at a higher risk of cardiovascular diseases and mortality in later life. CMR during exercise provides superior high resolution image quality and can produce 3D images of all the cardiac chambers. Several small studies have used CMR to obtain more detailed insight into cardiac adaptations to exercise and showed differences in cardiac response to exercise in diseased and non-diseased populations [16–18]. Combined with CMR, Isometric handgrip exercise is the most feasible physical stressor as it allows scanning without losing image quality due to movement artefacts [19].

Therefore, in a population-based prospective cohort study from early pregnancy onwards, we performed a cardiovascular stress test induced by isometric handgrip exercise combined with detailed measurements of the cardiovascular system. The objectives of this study were to develop a Cardiac Magnetic Resonance imaging (CMR) exercise study protocol using an isometric handgrip exercise in children, to demonstrate the feasibility and reproducibility of this protocol in a population-based cohort and to evaluate the cardiovascular response to exercise in a low-risk pediatric population.

## Methods

### Study design and subjects

**Fig. 1:**
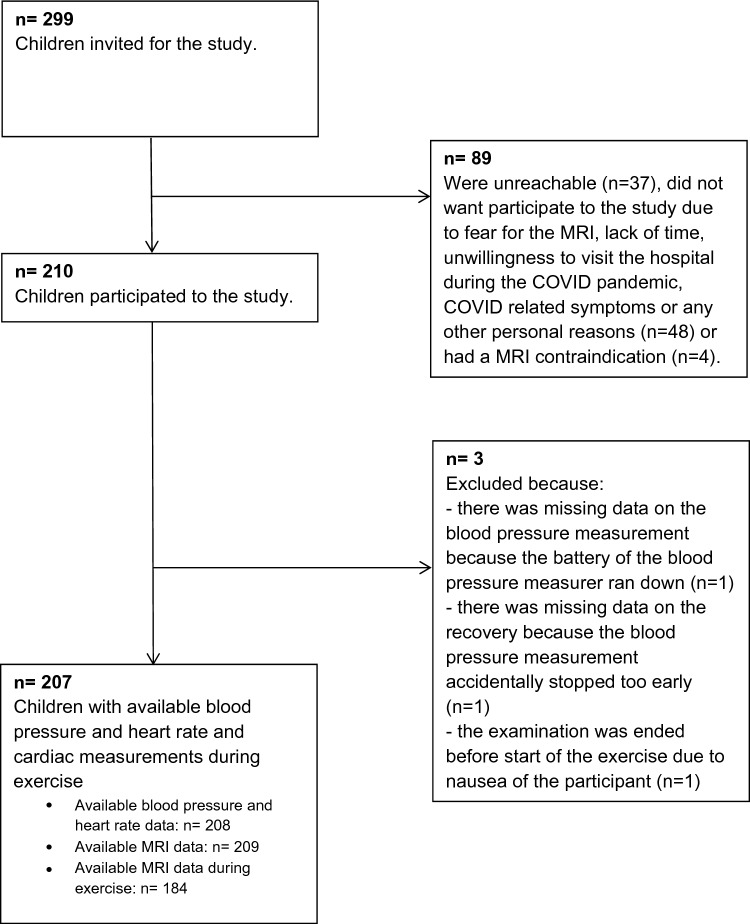
Flowchart of the study participants

This study was nested in the Generation R Study, a population-based prospective cohort study from early pregnancy onwards in Rotterdam, the Netherlands [20]. Approval for the study was obtained from the Medical Ethical Committee of Erasmus MC University Medical Center Rotterdam, The Netherlands. Written consent was obtained from all participants. In total, 8879 pregnant women were enrolled between 2001 and 2005. Of these mothers, a selected subsample of 1184 Dutch mothers had a known gestational age based on the last menstrual period, gave birth to a singleton live born child and had children with detailed assessments of fetal growth from first trimester onwards, postnatal growth and cardiovascular development until late childhood. We invited a random subgroup of 299 children from this sample to participate in the cardiovascular exercise test, of which 210 children visited our research center. The main reasons for non-participation were claustrophobia, a full school schedule or concerns regarding research center visits during the COVID-19 pandemic. After exclusion of children with missing blood pressure data during rest, peak exercise or recovery (n = 3), 207 children were available for analysis (Fig. 1). Mean age ± standard deviation (SD) of the children was 16.2 ± 0.7 years. Based on a priori hypotheses, we oversampled children who were overweight or obese according to the Cole criteria at previous research visits [21] and children born small for their gestational age (sex and gestational age adjusted birth weight below the 10th percentile in the study cohort) or preterm (born < 37 weeks of gestation) for future studies in higher risk groups.

### Handgrip exercise

**Fig. 2:**
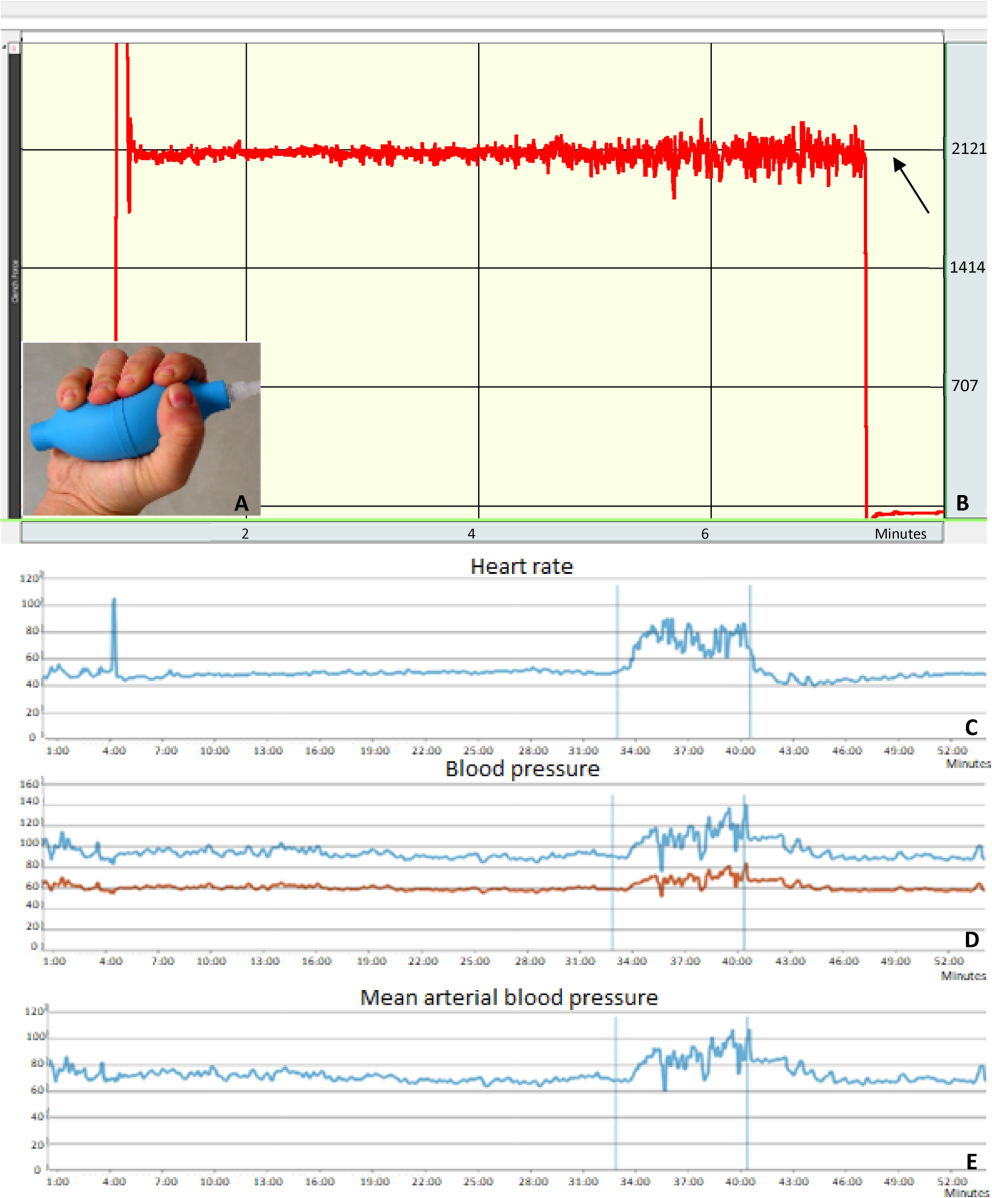
Handgrip exercise force, heart rate and blood pressure monitoring. Image of the clench force bulb that was used for the isometric handgrip exercise (**A**) and an example of the output of a handgrip exercise performed by a participant in kgf/m^2^ (**B**). The upper horizontal black line (arrow) in the figure corresponds to the 40% of the maximal voluntary contraction of the participant. **C**–**E**. Examples of a heart rate measurement in beats per minute (**C**), systolic (blue) and diastolic (red) blood pressure in mmHg (**D**) and mean arterial pressure in mmHg (**E**). The first vertical blue line corresponds to the start of the exercise and the second vertical blue line to the end of the exercise

This study was conducted in the Generation R MRI research center in the Erasmus University Medical Center, Rotterdam, Netherlands. To provoke a cardiovascular stress reaction, we used a sustained isometric handgrip exercise. Earlier studies have shown that a sustained handgrip of 30% to 40% of the maximal voluntary contraction (MVC) for 7 min is a feasible exercise that leads to a substantial increase in heart rate and blood pressure [19]. To measure the MVC, all participants were tested in the seated position with their elbow flexed at 90°. Maximal isometric voluntary contraction of the dominant hand was measured three times using the calibrated handgrip dynamometer SS56L hand clench force bulb, which consists of a soft plastic hand bulb which facilitated squeezing of the bulb with negligible body movement (Fig. 2A). Data on contraction force was analyzed by a corresponding software (Biopac Systems Inc., Santa Barbara, CA). If the trial measurements were not within 10% of each other, additional trials were performed until a consistent plateau was reached. The highest of the three values (kgf/m^2^) was defined as the subject’s maximal voluntary contraction (MVC). 30% and 40% MVC were calculated and taken forward to the cardiovascular exercise test during CMR. We used the same MRI‐compatible hand bulb, when the participant was positioned in the MRI scanner to conduct the handgrip exercise for the cardiovascular exercise test during CMR. Based on previous studies using a similar isometric handgrip exercise protocol, each volunteer was asked to sustain a grasp on the handgrip at 30–40% MVC with the dominant hand for a period of 7 min during which the cardiovascular exercise response to this exercise was measured by continuous heart rate and blood pressure monitoring and repeated CMR scans. The monitoring software continuously displayed the handgrip force induced by the participant (Fig. 2B). An MRI-compatible standalone mirror above the participant’s eyes enabled the participant to look outside the scanner in the direction of a beamer, located behind the scanner, on which the real-time force line was presented in the graph. The individual target force of 40% MVC was shown in the graph to facilitate motivating the participant to reach the 40% MVC with a minimum of 30% MVC. Besides the visual feedback, the operators guided and motivated the participant vocally.

### Continuous heart rate and blood pressure measurements during the cardiovascular exercise test

We obtained heart rate and blood pressure measurements during rest, exercise and recovery [5, 6, 22–25]. Heart rate was continuously measured during the cardiovascular exercise test by the CareTaker device (Empirical Technologies Corporation, Charlottesville, Virginia). This is a physiological sensing system, which consists of a finger cuff and a pressure line that pneumatically telemeters the pulsations to an electric pressure sensor outside the scanning room, which converts the pressure pulsations. Systolic blood pressure, diastolic blood pressure and mean arterial pressure were measured with the same CareTaker device (Fig. 2C–E**)**.

We converted the continuous measurements to mean measurements of heart rate and blood pressure for predefined time-intervals [26, 27]. We calculated the mean of 5 min rest before the start of the cardiovascular exercise test and mean of each minute of the 7-min exercise. The 7th minute of the exercise was defined as peak exercise. To measure the cardiovascular recovery reaction, we calculated the mean of each 30 s in the first two minutes after exercise cessation and at the 5th minute after exercise cessation.

### CMR measurements during the cardiovascular exercise test

Image acquisition was performed on a 3 T clinical MRI scanner (Discovery MR750w, GE Healthcare, Milwaukee, WI, USA) using a small torso 16ch body AA coil in combination with the table embedded coil array. The protocol included breath-hold balanced steady-state free precession (SSFP) cine imaging and 2D phase contrast images. First, 2D phase contrast images were acquired during breath hold at the level of the aortic root perpendicular to the aorta with the following parameters: FOV 360 × 288 mm, slice thickness 6 mm, matrix size 192 × 160, flip angle 20°, TR 6.0 ms, TE 3.8 ms, NEX 1, ASSET 2, VENC 200 cm/s, views per segment 6 and 30 reconstructed cardiac phases. Thereafter, both at rest and exercise (starting at 2 min), standard retrospective ECG-gated short axis SSFP images were obtained during repeated end-expiratory breath holds with coverage from base to apex of the ventricles. Scan parameters were: 2 slices per breath-hold, slice thickness 8 mm, inter-slice gap 2 mm, TR 3.1 ms, TE 1.5 ms, flip angle 45°, NEX 0.5, ASSET 2, field of view (FOV) 360 × 288 mm, acquired matrix 128 × 180 with 30 reconstructed phases per cardiac cycle. Furthermore, free-breathing high temporal resolution 2D phase contrast images of the ascending and descending aorta were acquired at the level of the pulmonary trunk at rest for pulse wave velocity measurements of the aorta. Scan parameters: FOV 252 × 360 mm, slice thickness 6 mm, matrix size 160 × 128, flip angle 20°, TR 5.0 ms, TE 3.1 ms, NEX 2, VENC 180 cm/s, views per segment 1 and 100 reconstructed cardiac phases. To determine the length of the aortic segment between the two aortic levels, sagittal angulated 3D SPGR images were acquired in a single breath hold (scan parameters: FOV 360 × 252 mm, slice thickness 3.4 mm, TR 1.7 ms, TE 0.8 ms, matrix size 100 × 192, NEX 4, flip angle 2°). Finally, also SSFP images were acquired at the level of the pulmonary trunk for aortic distensibility (FOV 360 × 360 mm, slice thickness 6 mm, TR 3.0 ms, TE 1.1 ms, flip angle 40°, NEX 1, ASSET 2, acquired matrix 160 × 224 with 30 reconstructed phases per cardiac cycle). Image analyses were performed by artificial intelligence-based automated analyses and were manually post-processed by two students. One student measured all ventricular outcomes, the second student measured aortic outcomes. The student measuring ventricular outcomes used software from Medis Medical Imaging Systems bv Leiden, The Netherlands: Medis Suite (v.3.2.60.6Q), QMass (v.8.1.98.2) and Qflow (v.8.1.98.2) and the other student used MASS(R) MR Analytical Software System, Leiden, The Netherlands (v. 2021). The analysis were performed according to the guidelines of the Society for Cardiovascular Magnetic Resonance (SCMR) [28]. Both students were trained and closely supervised by imaging CMR specialized cardiologist with > 20 years of experience (AH).

**Fig. 3:**
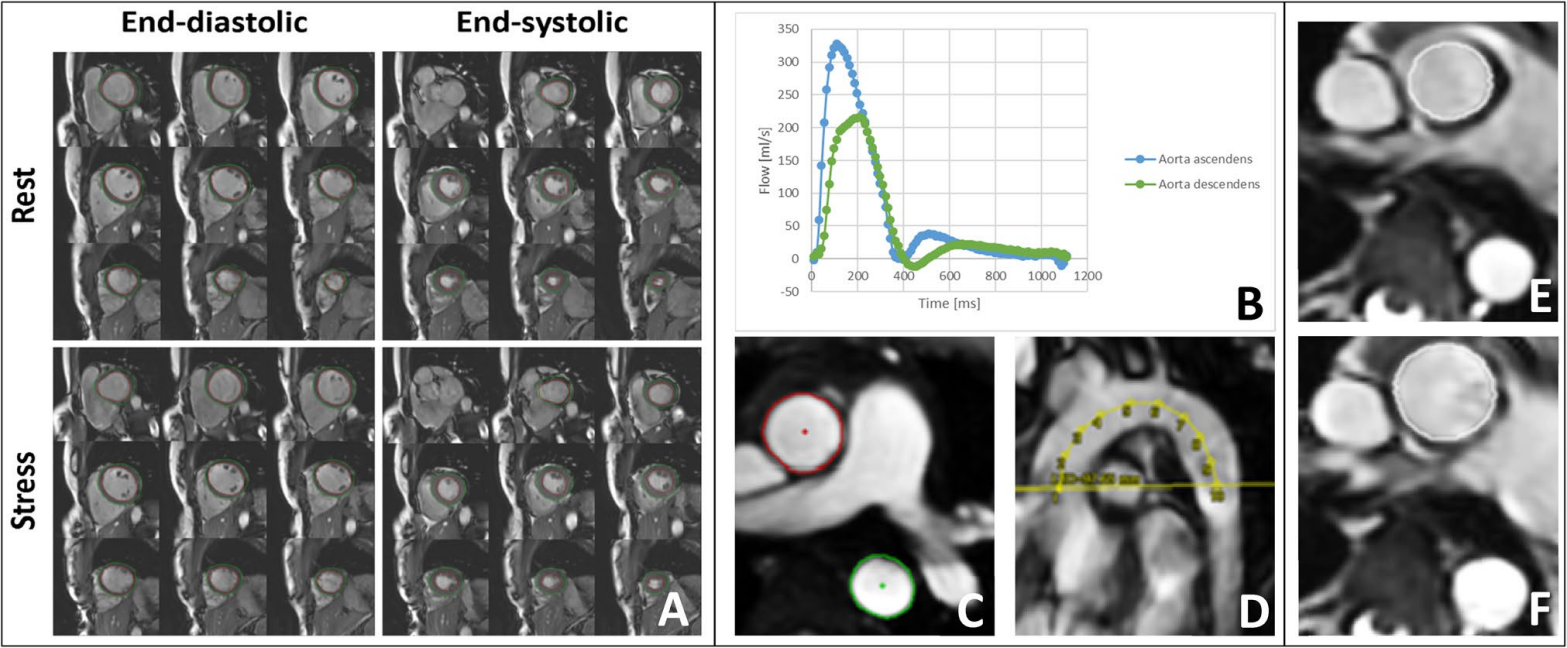
Example of the cardiac MRI images obtained during rest and exercise. **A** Left ventricle chamber quantification in short axis slices that cover the whole left ventricle. The left ventricle endocardium and left ventricle epicardium are delineated in systole and diastole, during rest and during exercise. **B**–**D**. Measurement of the pulse wave velocity (PWV). The contours of the ascending and descending aorta were drawn to measure the blood flow in ml/s (**B**, **C**). PWV was calculated as the ratio of distance Δx per time Δt, where Δx is the length of the aortic segment measured on the MR image data along the centerline (**D**), and Δt is the time duration needed for the pulse wave velocity to travel that length through the aorta. To calculate the Δt, we identified the time point of the maximum flow upslope of the ascending aorta and the maximum flow upslope of the descending aorta and calculated the Δt between these two time points in ms. **E**–**F** Calculation of the aortic distensibility. The minimum (**E**) and maximum (**F**) ascending aortic cross-sectional areas were measured. Aortic distensibility (10^–3^ mm Hg^−1^) was calculated using the following calculation: (maximum area – minimum area)/(minimum area × ΔP) × 1000, where ΔP is the brachial pulse pressure in mmHg

On the cine short-axis stack we measured left ventricular mass (LVM), left ventricular end-diastolic and end-systolic volume (LVEDV and LVESV respectively) and calculated stroke volume and ventricular ejection fraction (LVEF). Automatic segmentation was performed by delineation of the left endocardial and epicardial borders and then manually adjusted (Fig. 2A). Papillary muscles and trabecular tissue were included within the ventricle volumes [5, 29]. Additionally, left ventricular mass–volume ratio (LMVR) was calculated as LVM divided by LVDV. Cardiac output was calculated by multiplying stroke volume with heartrate. For calculation of cardiac output during exercise, mean heart rate measurements during the 7th minute of exercise were used. Aortic pulse wave velocity (PWV) and distensibility were measured using MASS(R). PWV was calculated as the ratio of distance Δx per time Δt, where Δx is the length of the aortic segment measured on the MR image along the centerline, and Δt is the time duration needed for the pulse wave to travel that length through the aorta. To calculate the Δt, we identified the maximum upslope of the velocity curve and calculated the time difference between the moment of the maximum upslope of the velocity curve in the ascending and descending aorta (Fig. 3B–D). For the calculation of the aortic distensibility, minimum and maximum ascending aortic cross-sectional areas were identified (Fig. 3E, F). Aortic distensibility (10^–3^ mm Hg^−1^) was calculated using the following calculation: (maximum area – minimum area)/(minimum area × ΔP) × 1.000, where ΔP is the difference between systolic and diastolic brachial pulse pressure in mmHg at time of the distensibility CMR scan.

Images of 30 randomly selected participants were analyzed twice by the same student to obtain intra-observer reproducibility. Both students performed their offline measurements twice, with an interval of two weeks to prevent recall bias. After the first reproducibility set, we observed a scope for improvement in the reproducibility of the distensibility measurements. The student was again trained by the same cardiologist (AH). After this training, the reproducibility test was repeated in another 30 random participants. All images were assessed for quality by the students. If there were any doubts on image quality, images were assessed by the cardiologist (AH). If images did not have enough quality, images were excluded from the analysis.

### Statistical analysis

First, differences in heart rate and blood pressure measurements between rest and exercise were calculated using a paired sample T-test. Differences in subject characteristics between boys and girls were assessed by Student's t-tests for independent samples for continuous variables and chi-square tests for proportions. Second, we examined reproducibility for all CMR measurements following Bland and Altman analyses [30, 31]. We plotted the measurements with the line of equality to explore the degree of agreement [30, 31]. Intraclass correlation coefficients (ICC) with a 95% confidence interval and the coefficients of variation (CV) were calculated for each measurement to evaluate consensus within the observer [30, 31]. Intra-observer variability was quantified calculating the mean difference in percentage measurement error with the 95% limits of agreement (mean difference [%] ± 1.96 SD) for all CMR measurements in rest and during exercise. Finally, differences in cardiac measurements between rest and exercise were examined using a paired sample T-test. The analyses were performed using the Statistical Package of Social Sciences version 24.0 for Windows (SPSS Inc., Chicago, IL, USA).

## Results

### Subject characteristics

Table 1 shows the population characteristics. Mean age was 16 years and 54% were boys. Mean resting systolic blood pressure was 117 ± 11 mmHg, mean resting diastolic blood pressure was 69 ± 8 and mean resting heart rate was 66 ± 9 beats per minute (bpm). Median LVM was 86 (95% range: 61–132)grams, mean resting LVEDV 172 ± 31 ml, resting LVESV 79 ± 16 ml, resting stroke volume 94 ± 18 ml/heartbeat, resting LVEF 54% ± 4, LMVR 0.51 ± 0.05, CO 6 ± 1.2 l/min, mean aortic distensibility was 10.8 ± 3.010^−3^ mmHg and median PWV was 3.9 (95% range: 2.0–9.3)m/s. All participants were motivated during the exercise and completed the entire 7 min exercise protocol of a consistent handgrip exercise at 30–40% MVC. Mean contraction during the cardiovascular exercise test was 2055 ± 423kgf/m^2^, which was 33.4% of the mean MVC. Mean contraction during the exercise was higher for boys than girls (p < 0.05).

**Table 1:** Characteristics of the study participants (n = 207)

	Total (n = 207)	Girls (n = 96)	Boys (n = 111)	P-value
Child characteristics				
Age, mean (SD), years	16.2 (0.7)	16.3 (0.6)	16.2 (0.7)	0.09
Dominant hand, n right (%)	192 (92.8)	90 (93.8)	102 (91.9)	0.76
Maximal voluntary contraction, mean (SD), kgf/m^2^	6162 (1610)	5575 (1076)	6669* (1816)	< 0.001
Mean contraction during exercise, mean (SD), kgf/m^2^	2055 (423)	1894 (296)	2196.8 (467)*	< 0.001
Resting systolic blood pressure, mean (SD), mmHg	116.7 (11.9)	113.6 (11.1)	119.3 (12.0)*	< 0.001
Resting diastolic blood pressure, mean (SD), mmHg	68.9 (8.5)	70.6 (8.2)	67.5 (8.5)	0.02
Resting mean arterial blood pressure, mean (SD), mmHg	84.2 (8.8)	84.9 (8.4)	83.6 (9.1)	0.29
Resting heart rate, mean (SD), bpm	67 (9)	68 (9)	66 (9)	0.15
CMR measurements				
Left ventricular mass, median (95%), gram	86 (61–132)	77 (58–95)	101 (67–133)*	< 0.001
Left ventricular mass/BSA, median (95%), gram/2	49 (35–67)	44 (5)	54 (7)*	< 0.001
Left ventricular end-diastolic volume at rest, mean (SD), ml	172 (31)	156 (19)	187 (33)*	< 0.001
Left ventricular end-diastolic volume at rest/BSA, mean (SD), ml/m2	96 (11)	90 (9)	101 (11)*	< 0.001
Left ventricular end-systolic volume at rest, mean (SD), m2	79 (16)	71 (10)	86 (17)*	< 0.001
Left ventricular end-systolic volume at rest /BSA, mean (SD), ml/m2	44 (7)	41 (5)	47 (7)*	< 0.001
Stroke volume, mean (SD), ml/heart beat	94 (18)	85 (13)	103 (17)*	< 0.001
Stroke volume/BSA, mean (SD), ml/heart beat/m2	52 (7)	49 (6)	55 (6)*	< 0.001
Left ventricular ejection fraction at rest, mean (SD), %	54 (4)	54 (4)	55 (5)	0.73
Left ventricular mass–volume ratio, mean (SD)	0.51 (0.05)	0.49 (0.04)	0.53 (0.05)*	< 0.001
Cardiac output, mean (SD), l/min	6.2 (1.2)	5.7 (1.0)	6.6 (1.1)*	< 0.001
Cardiac index, mean (SD), l/min/m2	3.5 (0.6)	3.3 (0.5)	3.6 (0.6)*	< 0.001
Aorta distensibility, mean (SD), 10^−3^ mmHg	11 (3)	12 (3)	10 (2)*	< 0.001
Regional area change (RAC), mean (SD), %	33 (5)	33 (5)	33 (5)	0.26
Pulse wave velocity, median (95%) m/s	3.9 (2.0–9.3)	3.8 (2.0–9.8)	4.0 (2.0–9.3)	0.89

Values are observed data and represent means (SD), medians (95% range) or numbers of subjects (valid %). Differences in subject characteristics between the boys and girls were evaluated using assessed by Student’s t-tests for independent samples for continuous variables and chi-square tests for proportions

*P-value < 0.05 for difference between girls and boys

### Heart rate and blood pressure measurements during the cardiovascular exercise test

**Fig. 4:**
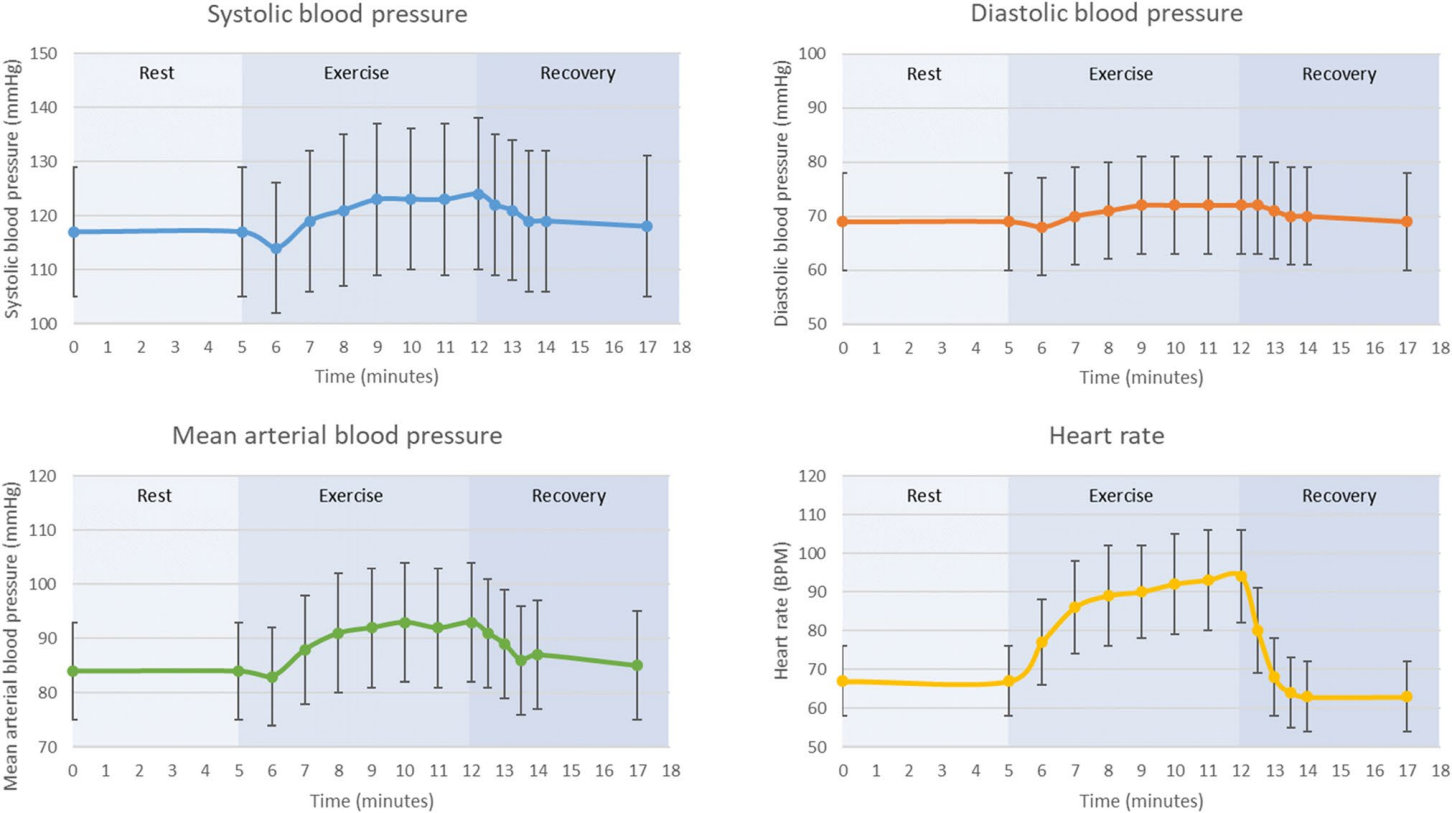
Blood pressure and heart rate response during rest, exercise and recovery. Mean measurements of systolic blood pressure, diastolic blood pressure, mean arterial blood pressure and heart rate during rest, exercise and recovery over time (minutes). Time points were: mean of 5 min rest before the start of the cardiovascular exercise test, mean of each minute of the 7-min exercise, mean of each 30 s in the first two minutes after exercise cessation and the mean of the 5th minute after exercise cessation

Table 2 shows the summarized changes in cardiovascular measurements in response to the exercise test from rest to peak exercise and to the 5th minute of the recovery. In the total group, mean heartrate increased during exercise with 42.6% ± 20.0, systolic blood pressure with 6.4% ± 7.0, diastolic blood pressure with 5.4% ± 6.1 and mean arterial blood pressure with 11.0% ± 8.3 (all p-values < 0.05). During recovery, heart rate declined by 32.4% ± 9.9 from peak exercise to the 5^th^ minute after cessation, systolic blood pressure declined by 4.9% ± 7.0, diastolic blood pressure by 4.3% ± 6.1 and mean arterial blood pressure by 8.4% ± 7.8. Boys had a higher systolic blood pressure during rest and recovery and a lower increase in systolic blood pressure in response to exercise. Girls had a higher diastolic blood pressure during rest and exercise (all p-values < 0.05). Mean blood pressure and heart rate patterns during rest, exercise and recovery are shown in Fig. 4. Supplemental Table 1 shows heart rate and blood pressure values at each specified time point of the cardiovascular exercise test.

**Table 2:** Differences in cardiovascular measurements between rest and peak exercise

	Rest	Exercise***	Mean difference rest to exercise*	Recovery
Heart rate, mean (SD), bpm				
Total	66 (9)	94 (12)	27 (11)	63 (9)
Girls	67 (8)	95 (12)	28 (13)	65 (9)
Boys	65 (9)	92 (12)	27 (11)	62 (8)
Systolic blood pressure, mean (SD), mmHg				
Total	117 (12)	124 (14)	7 (8)	119 (13)
Girls	113 (11)	122 (15)	9 (8)	115 (12)
Boys	119 (12)**	125 (13)	6 (8)**	123 (13)**
Diastolic blood pressure, mean (SD), mmHg				
Total	69 (8)	72 (9)	4 (4)	70 (9)
Girls	70 (8)	74 (8)	4 (4)	71 (8)
Boys	68 (8)**	70 (9)**	3 (4)	69 (9)
Mean arterial blood pressure, mean (SD), mmHg				
Total	84 (9)	93 (11)	9 (7)	87 (10)
Girls	85 (8)	94 (10)	10 (7)	86 (9)
Boys	83 (9)	92 (11)	9 (7)	87 (10)
Left ventricular end-diastolic volume/BSA, mean (SD), ml/m2				
Total	96 (11)	101 (15)	5 (10)	Na
Girls	90 (9)	94 (12)	4 (10)	Na
Boys	101 (11)**	107 (14)**	5 (10)	Na
Left ventricular end-systolic volume/BSA, mean (SD), ml/2				
Total	44 (7)	48 (10)	4 (7)	Na
Girls	41 (5)	45 (9)	4 (7)	Na
Boys	47 (7)**	51 (10)**	4 (6)	Na
Stroke volume/BSA, mean (SD), ml/heart beat/m2				
Total	52 (7)	52 (9)	< 1 (7)	Na
Girls	49 (6)	49 (8)	< 1 (7)	Na
Boys	55 (6)**	56 (9)**	< 1 (8)	Na
Left ventricular ejection fraction, mean (SD), %				
Total	54 (4)	52 (6)	− 2 (5)	Na
Girls	54 (4)	52 (6)	− 2 (5)	Na
Boys	54 (5)	52 (6)	− 2 (5)	Na
Left ventricular ejection fraction relative to heartrate, mean (SD) %/bpm				
Total	0.8 (0.1)	0.6 (0.1)	− 0.3 (0.1)	Na
Girls	0.8 (0.1)	0.6 (0.1)	− 0.3 (0.1)	Na
Boys	0.8 (0.1)	0.6 (0.1)	− 0.3 (0.1)	Na
Cardiac index, mean (SD), l/min/m2				
Total	3.5 (0.6)	4.9 (1.0)	1.5 (0.9)	Na
Girls	3.3 (0.5)	4.6 (0.9)	1.3 (0.9)	Na
Boys	3.6 (0.6)**	5.2 (1.0)**	1.5 (0.8)	Na

Cardiovascular measurements in rest, peak exercise, recovery (if applicable) and the difference between rest and peak exercise. Recovery was defined as the 5th minute of recovery after cessation of the exercise. All volumetric measurements (except for LVEF) were corrected for BSA

*Mean differences from rest to exercise were p < 0.001 for all values in the total group, girls and boys, except for stroke volume for which differences were not significant. Differences in cardiovascular outcomes between the boys and girls were evaluated using Student’s t-tests for independent samples

**P-value < 0.05 for difference between girls and boys

***For systolic, diastolic an mean arterial blood pressure and heart rate, the mean value in the 7^th^ minute of the exercise was used (peak exercise)

### Reproducibility CMR measurements

All 207 participants had good quality CMR measurements during rest and n = 184 during exercise (89%). Most excluded scans had a low quality because of breathing artefacts or failed ECG-triggering. Intra-observer ICCs for all measurements in rest and exercise were excellent (> 0.89) with CVs below 11%, except for LVEF for which we observed lower ICCs (0.69 in rest and 0.63 during exercise) (Supplemental table S2). Intra-observer analyses showed that all cardiac and aorta measurements lie in close proximity and evenly scattered around the lines of equality (Supplemental figure S1). The mean differences for all measurements in rest and exercise were below 8.1%, with limits of agreement from − 35.2% to 28.9%. For distensibility, we observed an excellent reproducibility with an ICC of 0.99 and a mean CV of 2 after a more extensive second training of the grading student (Supplemental table S2).

### CMR measurements during the cardiovascular exercise test

During exercise, LVEDV/BSA increased from 96 ± 11 to 101 ± 15 ml/m^2^, LVESV/BSA from 44 ± 7 to 48 ± 10 ml/m^2^ and cardiac index from 3.5 ± 0.6 to 4.9 ± 1.0 l/min/m^2^ whereas LVEF decreased from 54% ± 4 to 52%/ ± 6 (Table 2) (all p-values < 0.001). Change in LVEF was independent of heartrate. Corresponding percentage changes were 5.0% ± 10.6 for LVEDV, 10.1% ± 15.9 for LVESV, 42.2% ± 27.7 for cardiac output and − 4.0% ± 9.5 for LVEF. Stroke volume did not change significantly in response to exercise. Boys had a higher LVEDV, LVESV, stroke volume and cardiac output than girls both in rest and during exercise (all p-values < 0.05), both absolute and corrected for BSA. Supplemental table S3 shows the correlation coefficients between cardiac outcomes.

## Discussion

We performed a cardiovascular exercise test with detailed cardiovascular measurements in a pediatric population-based cohort study. We showed that a sustained handgrip exercise of 7 min at 30–40% MVC is a feasible exercise method in a healthy pediatric population to induce a cardiovascular exercise response. This handgrip exercise leads to significant increases in heart rate, blood pressure and cardiac left ventricular volumes.

Stress inducement by physical exercise requires major cardiovascular adaptations to maintain an adequate perfusion of the body. Cardiovascular exercise tests are widely used in clinical practice to reveal subtle cardiovascular pathology [3, 32–34]. In addition to common measurements, CMR during exercise tests improves the value of the cardiovascular exercise tests as it allows detailed assessment of the structural and functional cardiac response to exercise without geometric assumptions of the ventricles [33, 35, 36]. There are multiple methods available to induce the cardiovascular response to exercise. However, combined with CMR, cycling cannot be combined with long breath holds and a treadmill exercise is not feasible within the MRI. Isometric handgrip exercise is the most feasible physical stressor in combination with CMR as it allows scanning without losing image quality due to movement artefacts [19]. This is the first large pediatric population-based cohort study exploring the cardiovascular effects of an isometric handgrip exercise combined with detailed cardiovascular measurements [19].

The effects of isometric handgrip exercise on only simple measurements of the cardiovascular exercise response have been assessed by several small studies in children. In a population based study among 32 healthy children with a mean age of 15 years examining the cardiovascular exercise response to submaximal isometric handgrip, a sustained isometric handgrip exercise of 4 min at 25% MVC was performed, which led to an increase in heart rate of 25.7%, in systolic blood pressure of 12.7% and diastolic blood pressure of 24.6% [37]. In other small clinical pediatric studies among children aged 10 to 18 years ranging from n = 19 to n = 47, heart rate increased up to 18 bpm, systolic blood pressure increased by 15 mmHg and diastolic blood pressure by 16 mmHg, induced by an isometric handgrip exercise at varying intensities and durations from 30 s to 4 min [38–43]. As compared to these studies, our exercise protocol led to stronger effects on increases in heart rate, but lower effects on increases in blood pressure.

We observed a steep decline in heart rate and blood pressure after cessation of the exercise, and after 5 min cessation heartrate was lower and blood pressure was still higher than before exercising. In line with our findings, a study in 27 children aged 12 years measured the cardiovascular recovery reaction after a 3-min isometric handgrip exercise at 30%MVC and showed that the mean arterial pressure was slightly higher and heart rate was lower after a 3-min recovery than before exercising [38]. Differences between our study and previous studies may be explained by differences in exercise protocol and cardiovascular assessment. Compared to previous studies, our exercise protocol was much longer to ensure that all CMR images were made while the cardiovascular system was at a stress state. Also, in our study, participants were in a supine position and had to hold their breath for cardiac imaging. Systemic vascular resistance at the start of supine exercise is lower and increases less than during erect exercise, therefor blood pressure changes are lower during supine exercise [44, 45]. This may, at least partly, explain the differences between our results and those from previous studies. Thus, we showed that a sustained isometric handgrip exercise of 7 min at 30–40%MVC leads to a significant rise in heart rate and blood pressure, enabling the assessment of a cardiovascular exercise reaction by a simple, low-cost exercise.

In research settings, only few studies have investigated the cardiovascular stress response with exercise CMR in healthy populations and involved only adults. In the vast majority, an MRI compatible cycle or push–pull ergometer was used as exercise modality. A meta-analysis of 17 previous exercise CMR studies with a total of 226 healthy adult subjects, showed an increase in both heart rate and stroke volume in reaction to various forms of dynamic exercises (e.g. cycle, treadmill or custom made modality). The change in stroke volume was driven by a reduction in left ventricular end-systolic volume, with no change in left ventricular end-diastolic volume. Left ventricular ejection fraction slightly increased [46]. There are only a few studies with relatively small sample sizes, examining the cardiovascular reaction on an isometric handgrip exercise measured by CMR. A study exploring the cardiovascular response to an isometric handgrip exercise of 6–8 min at 30% MVC in 53 healthy subjects with a mean age of 45 years found an increase in stroke volume from 78 to 80 ml/heartbeat [35]. A study in 333 healthy subjects with a mean age of 53 years old explored the cardiovascular response to an isometric handgrip exercise of 3 min at 40% MVC measured by echocardiography. They found an increase in LVEDV, LVESV and stroke volume and a small decrease in ejection fraction [47]. This study showed that younger subjects had a much higher increases in LVESV and LVEDV. A study in 75 healthy participants aged 38.8 ± 10.9 years, examined the effects of an isometric bicep exercise at 35% of maximum biceps force on ventricular outcomes measured by CMR. This study also showed an increase in LVEDV and LVESV (6.0% and 20.8%, respectively), decrease in LVEF (-6.3%) and unchanged stroke volume in response to exercise [48]. In line with these studies in adult populations, we observed significant changes in LVEDV, LVESV, LVEF and cardiac output in response to isometric handgrip exercise. Stroke volume however remained unchanged. The small decrease in LVEF is in contrast with the effects of a dynamic exercise which increases LVEF [49]. This can be explained by the increase in systemic vascular resistance during isometric exercise, whilst it decreases in response to dynamic exercise [50]. As a result, isometric exercise causes a larger increase in afterload, which limits LV ejection through the force velocity relationship [26]. Of all good quality CMR measurements during rest, we were able to use 184 CMR scans (89%) of good quality obtained during exercise. Due to time restriction, we scanned two slices per breath-hold resulting in longer breath-holds of maximal 15 s which were not feasible for some of the participants during the exercise, leading to breathing artefacts. Also, the ECG triggering of the scanner failed in some of the participants during the exercise causing low quality images which were not suitable for further analyses. We observed a good to excellent intra-observer reproducibility for the majority of volumetric and functional cardiac and aortic measurements obtained during rest and the cardiovascular exercise test [51]. Only for LVEF, reproducibility was moderate. To improve reproducibility, we repeated a part of the training and applied stricter rules for identifying the left ventricular basal slice, including the rule that the basal slice may be defined by at least 50% of the blood volume surrounded by myocardium [28]. Thus, our study shows that CMR during exercise is feasible in pediatric populations and leads to significant increases in LVEDV, LVESV and cardiac output with the strongest effect on LVEDV and LVESV. LVEF slightly decreased, in line with earlier research examining the effects of an isometric exercise.

We explored potential differences between girls and boys, as earlier studies describe different physical reactions to exercise according to sex [52]. In girls, systolic blood pressure increased more during exercise and decreased faster after cessation of the exercise compared to boys. This is in contrast with earlier pediatric and adult studies finding stronger effects of exercise on heart rate and blood pressure in men [47, 53] These differences may be due to the age of assesment and our exercise protocol. Further studies are needed to examine the differences in cardiovascular exercise reaction and explore the potential underlying mechanisms explaining differences in exercise response between boys and girls.

### Future research

The findings from our study offer great opportunities for future research examining the effects of early life exposures on childhood cardiovascular development as a sustained isometric handgrip exercise can reveal subtle functional adaptations of the cardiovascular system that may not be present at rest. These results could aid in finding better screening modalities to identify children that are at an increased risk of future cardiovascular diseases and mortality. These findings are also important from a clinical perspective as these findings provide insight into the potential to perform an isometric handgrip exercise during cardiac MR scanning to induce a cardiovascular exercise response when pharmacological stressors are contraindicated or undesirable. However, 11% of our CMR scans during exercise were not of sufficient quality for assessment and were excluded from our analysis. Newer CMR techniques allow for real-time free breathing scanning during exercise with preservation of the image quality [54]. A recent study even successfully developed a free-breathing and ECG free real-time cine for exercise CMR [55]. This offers great opportunities for future CMR exercise studies.

In the current study we measured PWV during rest. Future studies should include measurements of PWV during rest and exercise to obtain a more complete overview of the cardiovascular exercise response. These PWV measurement should include additional calculation methods, like the location specific flow-area method (PWV_QA_). This method correlates very well to the method used in the current study and allows for the calculation of regional differences in stiffness between the ascending and descending aorta [57]. Additionally by using phase contrast MRI-derived flow and area waveforms, wave intensity analysis can be performed to obtain a non-invasive measurement of the arterial wall stiffness without the use of breath holds [58]. More information on cardiac functioning during exercise could also be obtained by adding strain measurements to the analysis. This method differentiates between active and passive movement of myocardial segments and detects early subclinical signs of myocardial dysfunction [56, 59].

In this study we found a small decrease in LVEF, which is contrary to the effects of dynamic exercise. Future studies should compare an isometric handgrip exercise with dynamic exercise to explore which method is more effective in discovering small subclinical differences in cardiovascular exercise reaction.

## Conclusion

A sustained handgrip exercise of 7 min at 30–40% MVC is a feasible exercise in a healthy pediatric population, which leads to reproducible cardiovascular system measurements. This handgrip exercise leads to significant increases in heart rate, blood pressure, LVEDV, LVESV, and cardiac output. This approach offers great research opportunities for future population studies assessing cardiovascular health and dysfunction in pediatric populations.

### Supplementary Information

Below is the link to the electronic supplementary material.

Supplementary file1 (DOCX 447 KB)
