# Supplementary material for: Physical exercise and cardiovascular response: design and implementation of a pediatric CMR cohort study
Source: Int J Cardiovasc Imaging. 2023 Oct 6;39(12):2575–87. doi: 10.1007/s10554-023-02950-7 (PMC10691979; doi:10.1007/s10554-023-02950-7)
Supplement: Supplementary file 1 — Supplementary file1 (DOCX 447 KB) [file 10554_2023_2950_MOESM1_ESM.docx]

Supplementary material

**Physical exercise and cardiovascular response:**

**Design and implementation of a pediatric CMR cohort study**

*Running title: Isometric handgrip exercise CMR study in a pediatric population-based cohort*

Meddy N. Bongers-Karmaoui MD^1,2^, Alexander Hirsch MD PhD^3,4^, Ricardo P.J. Budde MD PhD^4^, Vincent W.V. Jaddoe MD PhD^1,2^, Romy Gaillard MD PhD^1,2^

1. The Generation R Study Group, Erasmus University Medical Center, Rotterdam, the Netherlands.
2. Department of Pediatrics, Sophia Children's Hospital, Erasmus University Medical Center, Rotterdam, the Netherlands.
3. Department of Cardiology, Erasmus University Medical Center, Rotterdam, the Netherlands.
4. Department of Radiology, Erasmus University Medical Center, Rotterdam, the Netherlands.

Corresponding author

Romy Gaillard, The Generation R Study Group, Erasmus University Medical Center, PO Box 2040, 3000 CA Rotterdam, The Netherlands (r.gaillard@erasmusmc.nl). Telephone number: 0031 10 704 3405

**Content**

**Table S1.** Haemodynamic changes during the cardiovascular stress test

**Figure S1.** Measurements plotted with line of equality for the measurement of all cardiac measurements.

**Table S2.** Intra-observer reproducibility outcomes for the cardiovascular magnetic resonance measurements.

**Table S3.** Correlation between all cardiovascular magnetic resonance measurements.

**Supplemental table 1.** Haemodynamic changes during the cardiovascular stress test

|  | **Total** | | | | **Girls** | | | | **Boys** | | | |
| --- | --- | --- | --- | --- | --- | --- | --- | --- | --- | --- | --- | --- |
|  | Systolic blood pressure (mmHg) | Diastolic blood pressure (mmHg) | Mean arterial pressure (mmHg) | Heart rate (BPM) | Systolic blood pressure (mmHg) | Diastolic blood pressure (mmHg) | Mean arterial pressure (mmHg) | Heart rate (BPM) | Systolic blood pressure (mmHg) | Diastolic blood pressure (mmHg) | Mean arterial pressure (mmHg) | Heart rate (BPM) |
| **Rest** |  |  |  |  |  |  |  |  |  |  |  |  |
| Mean of 5 minutes before exercise (sd) | 117(12) | 69 (9) | 84 (9) | 67 (9) | 114 (11) | 71 (8) | 85 (8) | 68 (9) | 11 (12)* | 68 (9)* | 84 (9) | 66 (9) |
| **Exercise** |  |  |  |  |  |  |  |  |  |  |  |  |
| 1^st^ minute exercise (sd) | 114 (12) | 68 (9) | 83 (9) | 77 (11) | 112 (12) | 70 (8) | 84 (9) | 77 (11) | 117 (12)* | 66 (9)* | 82 (10) | 77 (11) |
| 2^nd^ minute exercise (sd) | 119 (13) | 70 (9) | 88 (10) | 86 (12) | 117 (13) | 72 (8) | 89 (10) | 86 (13) | 120 (13)* | 68 (9)* | 87 (11) | 86 (11) |
| 3^rd^ minute exercise (sd) | 121 (14) | 71 (9) | 91 (11) | 89 (13) | 120 (13) | 73 (8) | 92 (10) | 90 (14) | 123 (14)* | 70 (10)* | 9 (12) | 89 (11) |
| 4^th^ minute exercise (sd) | 123 (14) | 72 (9) | 92 (11) | 90 (12) | 122 (13) | 75 (8) | 94 (9) | 92 (13) | 125 (14) | 70 (10)* | 91 (12) | 89 (11) |
| 5^th^ minute exercise (sd) | 123 (13) | 72 (9) | 93 (11) | 92 (13) | 122 (12) | 75 (8) | 94 (9) | 93 (13) | 125 (14) | 70 (10)* | 91 (12) | 91 (12) |
| 6^th^  minute exercise (sd) | 123 (14) | 72 (9) | 92 (11) | 93 (13) | 122 (14) | 75 (8) | 94 (10) | 94 (14) | 125 (13) | 70 (10)* | 91 (12) | 92 (12) |
| 7^th^  minute exercise (sd) | 124 (14) | 72 (9) | 93 (11) | 94 (12) | 122 (14) | 75 (8) | 94 (10) | 95 (13) | 125 (14) | 70 (10)* | 92 (11) | 92 (12) |
| **Recovery** |  |  |  |  |  |  |  |  |  |  |  |  |
| 1^st^ half minute after exercise (sd)† | 122 (13) | 72 (9) | 91 (10) | 80 (11) | 120 (13) | 74 (8) | 92 (9) | 80 (10) | 124 (13)* | 70 (9)* | 90 (11) | 79 (11) |
| 2^nd^ half minute after exercise (sd)† | 121 (13) | 71 (9) | 89 (10) | 68 (11) | 117 (13) | 72 (9) | 89 (10) | 69 (10) | 125 (13)* | 70 (9)* | 89 (11) | 68 (11) |
| 1^st^ half of the 2^nd^ minute after exercise (sd)† | 119 (13) | 70 (9) | 86 (10) | 64 (9) | 115 (12) | 71 (9) | 86 (9) | 65 (9) | 123 (13)* | 69 (9) | 87 (11 | 63 (9) |
| 2^nd^ half of the 2^nd^ minute after exercise (sd)† | 119 (13) | 70 (9) | 87 (10) | 63 (9) | 115 (12) | 71 (9) | 86 (9) | 64 (9) | 122 (13)* | 69 (9) | 87(11) | 63 (9) |
| 5^th^ minute after exercise (sd) | 118 (13) | 69 (9) | 85 (10) | 63 (9) | 114 (12) | 70 (9) | 85 (9) | 64 (9) | 121 (13)* | 68 (9) | 85 (10) | 62 (9) |

Table S1. Every value correspondents to the mean value of the blood pressure or heart rate of 60 seconds from the beginning to the end of the respective minute. Differences in cardiovascular outcomes between the boys and girls were evaluated using Student's t-tests for independent samples. *p<0.05
Variables with a † correspondents to a mean value of 30 seconds.
Sd: standard deviation

**Supplemental figure S1**. Measurements plotted with line of equality for the measurement of all cardiac measurements.


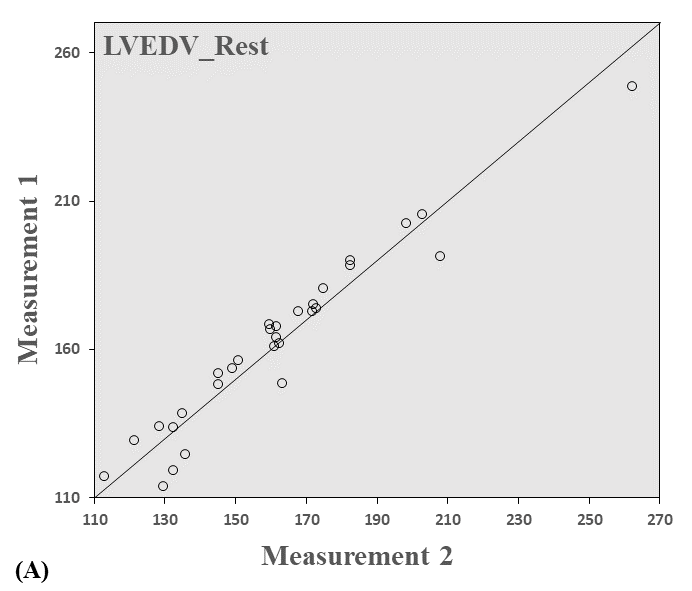

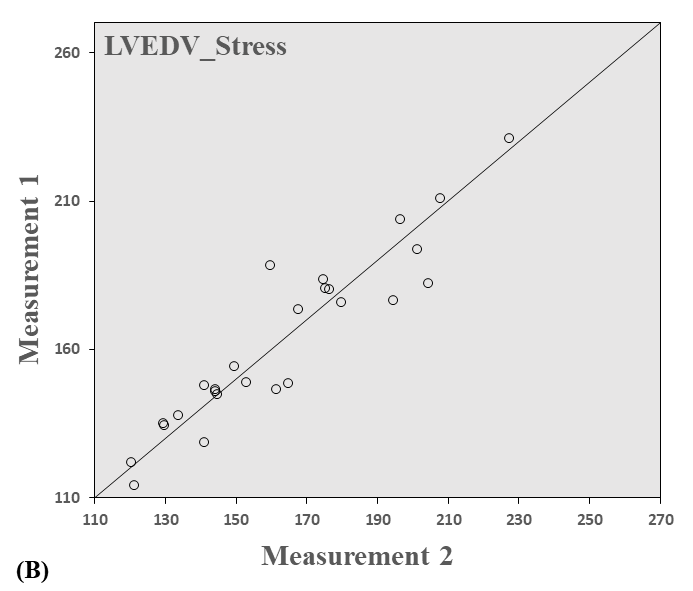


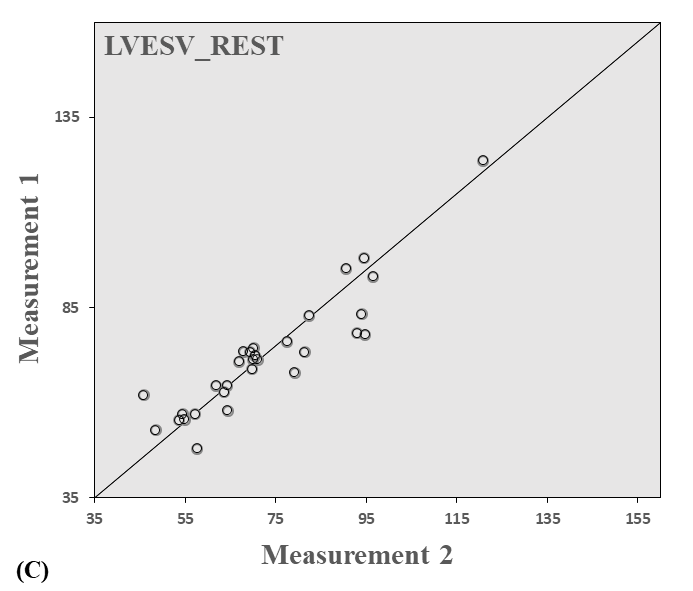

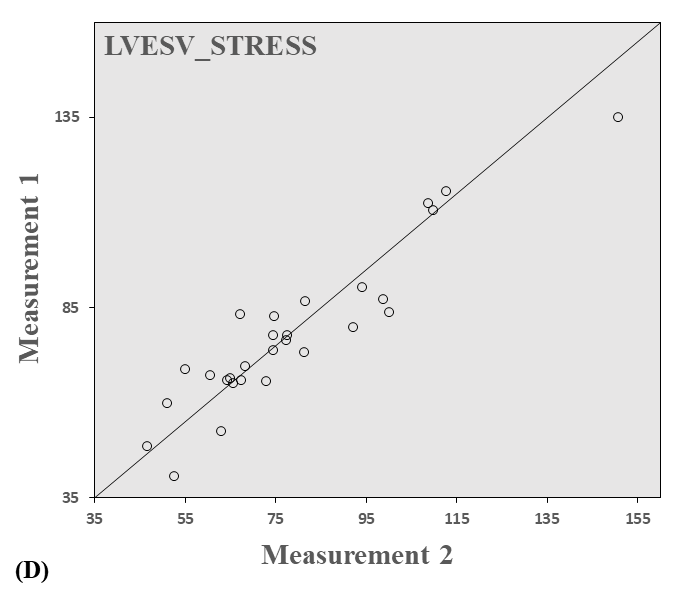


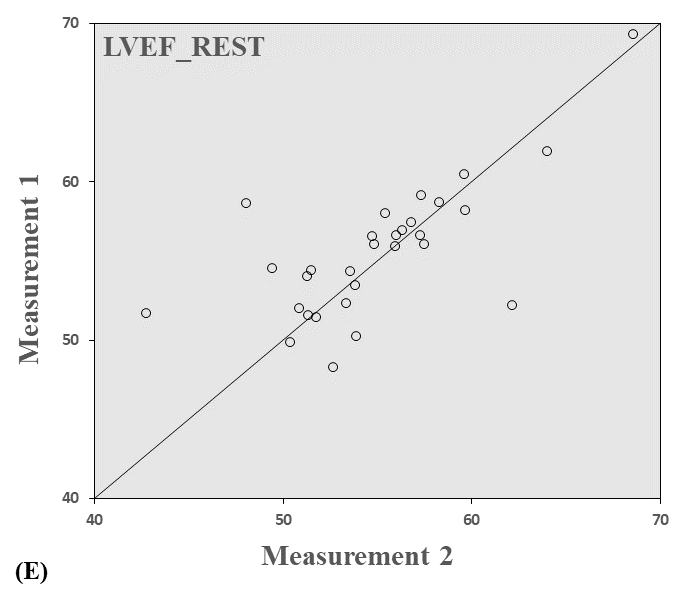

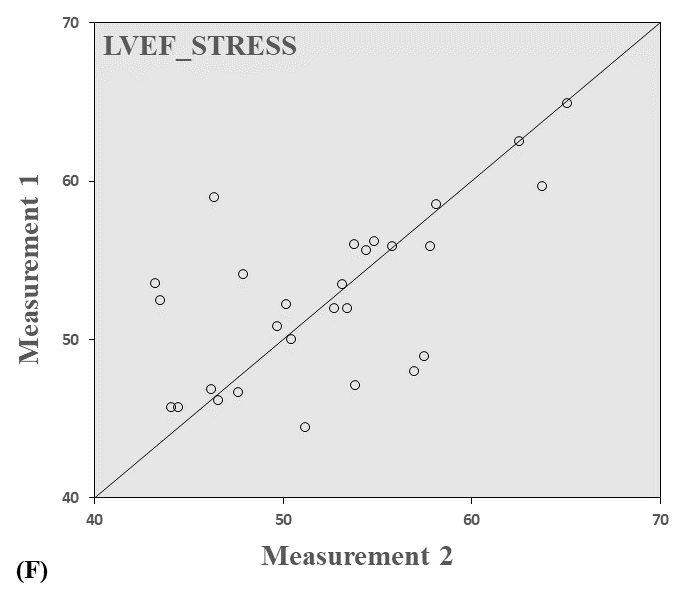


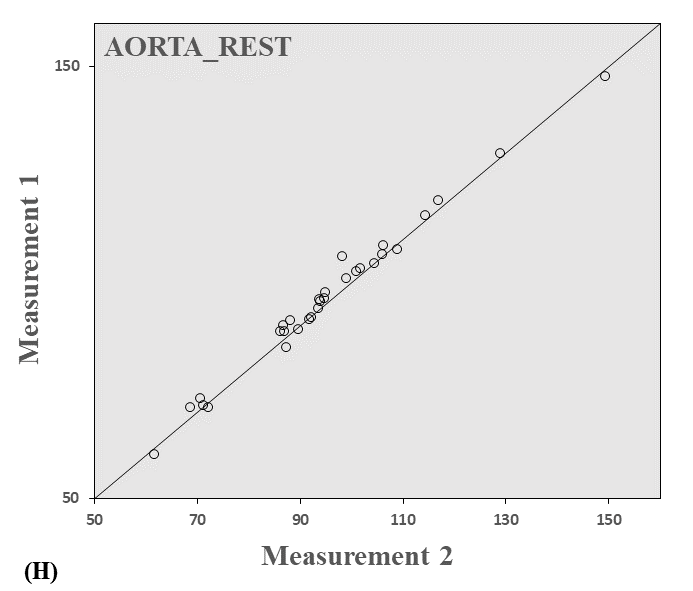

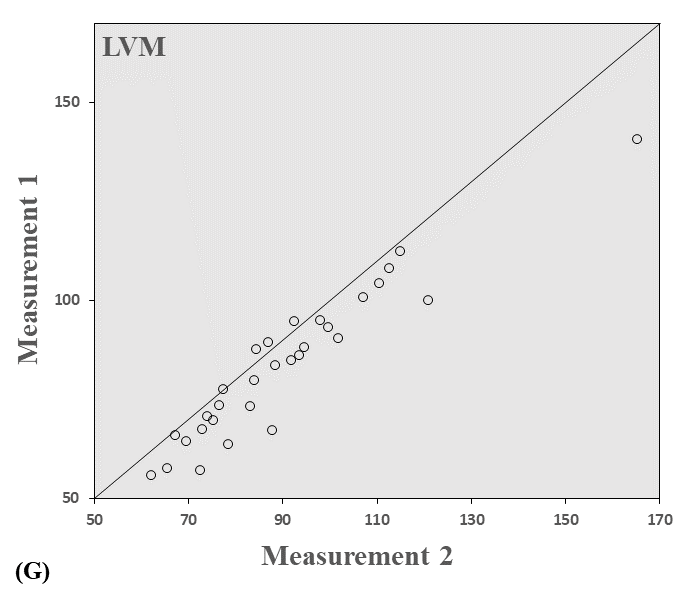

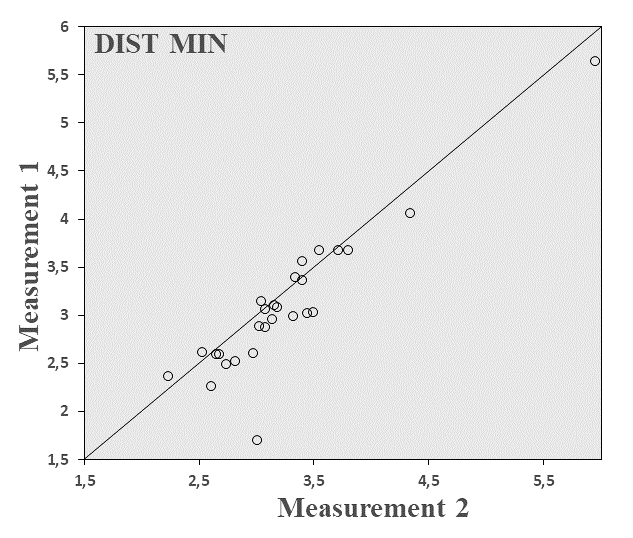


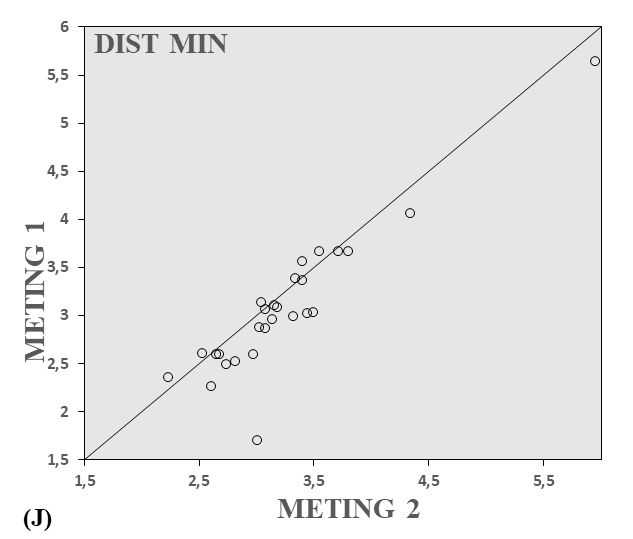

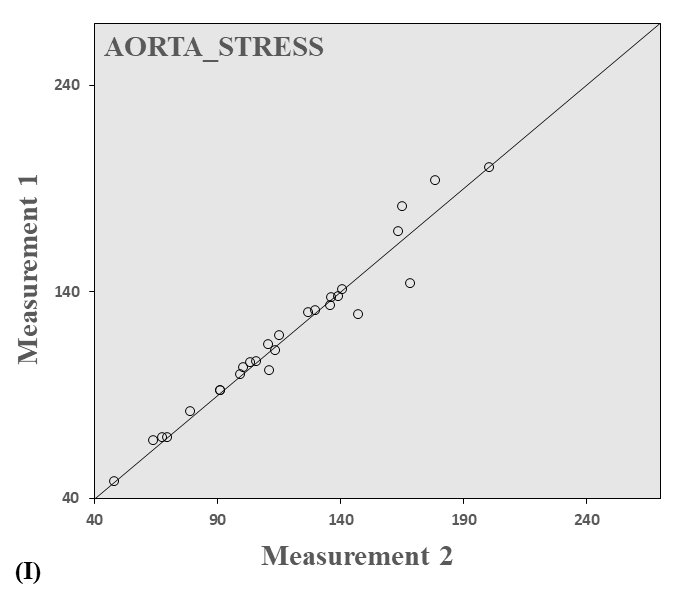

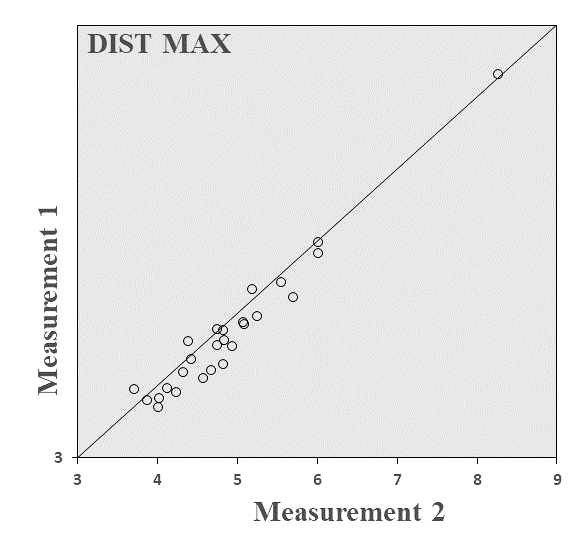

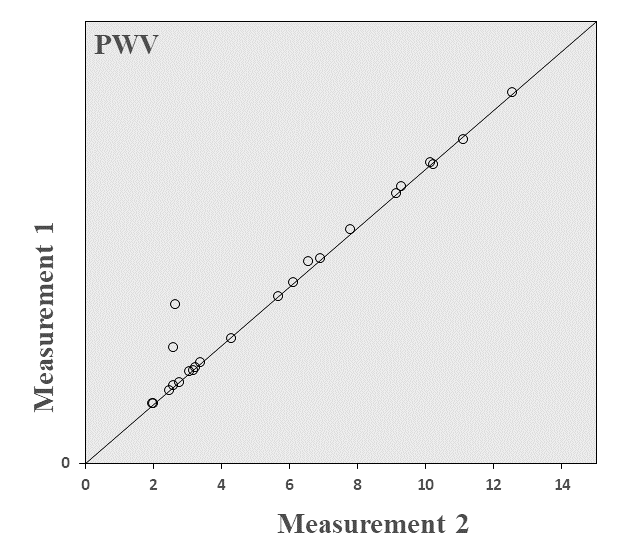


Figure S1. A)Left ventricular end-diastolic volume in rest , B) Left ventricular end-diastolic volume in stress, C) Left ventricular end-systolic volume in rest, D) Left ventricular end-systolic volume in stress, E) Left ventricular ejection fraction in rest, F) Left ventricular ejection fraction in stress, G) Left ventricular mass, H) Minimal aortic diameter for calculation of the distensibility, I) Maximal aortic diameter for calculation of the distensibility, J) Pulse wave velocity.

**Supplemental table S2.** Intra-observer reproducibility outcomes for the cardiovascular magnetic resonance measurements.

|  | CV | Mean difference % | Upper LOA | Lower LOA | ICC (95%) |
| --- | --- | --- | --- | --- | --- |
| LVEDV rest | 4.85 | 0.37 | -10.48 | 9.74 | 0.97 (0.93, 0.98) |
| LVEDV stress | 6.71 | 0.48 | -11.80 | 12.76 | 0.95 (0.90, 0.98) |
| LVESV rest | 9.46 | 0.76 | -18.68 | 20.20 | 0.91 (0.83, 0.96) |
| LVESV stress | 10.56 | 1.00 | -21.34 | 23.34 | 0.93 (0.85, 0.97) |
| LVEF rest | 6.61 | 1.16 | -14.72 | 12.40 | 0.69 (0.45, 0.84) |
| LVEF stress | 9.49 | 0.77 | -19.94 | 18.40 | 0.63 (0.34, 0.81) |
| LVM | 7.75 | 8.07 | -6.52 | 22.66 | 0.89 (0.40, 0.97) |
| DIST MIN | 9.13 | 6.27 | -16.39 | 28.93 | 0.89 (0.70, 0.96) |
| DIST MAX | 9.25 | 5.70 | -16.55 | 27.95 | 0.87 (0.67, 0.94) |
| DIST MIN* | 2.44 | 0.51 | -4.04 | 5.06 | 0.99 (0.99, 1.00) |
| DIST MAX* | 2.03 | 0.16 | -4.09 | 3.77 | 0.99 (0.99, 1.00) |
| PWV | 10.50 | 4.87 | -35.18 | 25.44 | 0.98 (0.96, 0.99) |

**Table S2.** Al values are measures of the intra-observer reproducibility. CV: coefficients of variation, LOA: limits of agreement, ICC: intraclass correlation coefficients with corresponding 95% confidence interval, LVEDV: Left ventricular end-diastolic volume, LVESV: Left ventricular end-systolic volume, LVEF: Left ventricular ejection fraction, LVM: Left ventricular mass, DIST MIN: Minimal aortic diameter for calculation of the distensibility, DIST MAX: Maximal aortic diameter for calculation of the distensibility, PWV: pulse wave velocity.
*Reproducibility outcomes in a new set with a second check of the contours by the same observer(n=30)

**Supplemental table S3.** Correlation between all cardiovascular magnetic resonance measurements

Table S3. Correlation between the cardiovascular magnetic resonance measurements measured with Pearson’s correlation coefficient. LVM: Left ventricular mass, LVEDV: Left ventricular end-diastolic volume, LVESV: Left ventricular end-systolic volume, LVEF: Left ventricular ejection fraction.

|  | LVEDV REST | LVEDV STRESS | LVESV REST | LVESV STRESS | LVEF REST | LVEF STRESS | LVM REST | STROKE VOLUME REST | STROKE VOLUME STRESS | CARDIAC OUTPUT REST | CARDIAC OUTPUT STRESS |
| --- | --- | --- | --- | --- | --- | --- | --- | --- | --- | --- | --- |
| LVEDV REST | Na | 0.810 | 0.888 | 0.728 | -0.157 | -0.166 | 0.826 | 0.888 | 0.653 | 0.562 | 0.374 |
| LVEDV STRESS | 0.810 | Na | 0.749 | 0.868 | -0.172 | -0.189 | 0.714 | 0.695 | 0.831 | 0.388 | 0.379 |
| LVESV REST | 0.888 | 0.749 | Na | 0.777 | -0.515 | -0.343 | 0.770 | 0.577 | 0.497 | 0.351 | 0.256 |
| LVESV STRESS | 0.728 | 0.868 | 0.777 | Na | -0.318 | -0.570 | 0.658 | 0.533 | 0.460 | 0.253 | 0.169 |
| LVEF REST | -0.157 | -0.172 | -0.515 | -0.318 | Na | 0.472 | -0.053 | 0.273 | 0.076 | 0.280 | 0.145 |
| LVEF STRESS | -0.166 | -0.189 | -0.343 | -0.570 | 0.472 | Na | -0.153 | 0.023 | 0.366 | 0.137 | 0.371 |
| LVM | 0.826 | 0.714 | 0.770 | 0.658 | -0.053 | -0.153 | Na | 0.772 | 0.569 | 0.443 | 0.336 |
| STROKE VOLUME REST | 0.888 | 0.695 | 0.577 | 0.533 | 0.273 | 0.023 | 0.772 | Na | 0.677 | 0.660 | 0.456 |
| STROKE VOLUME STRESS | 0.653 | 0.831 | 0.497 | 0.460 | 0.076 | 0.366 | 0.569 | 0.677 | Na | 0.450 | 0.593 |
| CARDIAC OUTPUT REST | 0.562 | 0.388 | 0.351 | 0.253 | 0.280 | 0.137 | 0.443 | 0.660 | 0.450 | Na | 0.508 |
| CARDIAC OUTPUT STRESS | 0.374 | 0.379 | 0.256 | 0.169 | 0.145 | 0.371 | 0.336 | 0.456 | 0.593 | 0.508 | Na |
